# Paulomycin G, a New Natural Product with Cytotoxic Activity against Tumor Cell Lines Produced by Deep-Sea Sediment Derived *Micromonospora matsumotoense* M-412 from the Avilés Canyon in the Cantabrian Sea

**DOI:** 10.3390/md15090271

**Published:** 2017-08-28

**Authors:** Aida Sarmiento-Vizcaíno, Alfredo F. Braña, Ignacio Pérez-Victoria, Jesús Martín, Nuria de Pedro, Mercedes de la Cruz, Caridad Díaz, Francisca Vicente, José L. Acuña, Fernando Reyes, Luis A. García, Gloria Blanco

**Affiliations:** 1 Departamento de Biología Funcional, Área de Microbiología, and Instituto Universitario de Oncología del Principado de Asturias, Universidad de Oviedo, 33006 Oviedo, Spain; UO209983@uniovi.es (A.S.-V.); afb@uniovi.es (A.F.B.); 2 Fundación MEDINA, Centro de Excelencia en Investigación de Medicamentos Innovadores en Andalucía, Avda, del Conocimiento 34, Parque Tecnológico de Ciencias de la Salud, E-18016 Granada, Spain; ignacio.perez-victoria@medinaandalucia.es (I.P.-V.); jesus.martin@medinaandalucia.es (J.M.); ndepedro@lifelength.com (N.d.P.); mercedes.delacruz@medinaandalucia.es (M.d.l.C.); caridad.diaz@medinaandalucia.es (C.D.); francisca.vicente@medinaandalucia.es (F.V.); 3 Departamento de Biología de Organismos y Sistemas, Área de Ecología, Universidad de Oviedo, 33006 Oviedo, Spain; acuna@uniovi.es; 4 Departamento de Ingeniería Química y Tecnología del Medio Ambiente, Área de Ingeniería Química, Universidad de Oviedo, 33006 Oviedo, Spain; luisag@uniovi.es

**Keywords:** paulomycins, *Micromonospora*, antitumor, Cantabrian Sea-derived actinobacteria

## Abstract

The present article describes a structurally novel natural product of the paulomycin family, designated as paulomycin G (**1**), obtained from the marine strain *Micromonospora matsumotoense* M-412, isolated from Cantabrian Sea sediments collected at 2000 m depth during an oceanographic expedition to the submarine Avilés Canyon. Paulomycin G is structurally unique since—to our knowledge—it is the first member of the paulomycin family of antibiotics lacking the paulomycose moiety. It is also the smallest bioactive paulomycin reported. Its structure was determined using HRMS and 1D and 2D NMR spectroscopy. This novel natural product displays strong cytotoxic activities against different human tumour cell lines, such as pancreatic adenocarcinoma (MiaPaca_2), breast adenocarcinoma (MCF-7), and hepatocellular carcinoma (HepG2). The compound did not show any significant bioactivity when tested against a panel of bacterial and fungal pathogens.

## 1. Introduction

Paulomycins are glycosylated natural products featuring a pauloate residue with pharmacological interest due to their antibiotic activities. Paulomycins A and B are antibiotics with very potent activity against Gram-positive bacteria (*Staphylococcus aureus* and *Bacillus cereus*) and are of therapeutic use in the treatment of gonococcal and *Chlamydia* infections [1]. Initially described in *Streptomyces paulus* [2] and later in *Streptomyces albus* J1074 [3], a series of paulomycins with various modifications at the two-carbon branched chain of paulomycose were subsequently isolated from these *Streptomyces* species [3,4,5]. The biosynthetic pathway of paulomycins is also the subject of active research [6,7], and no chemical synthesis has been reported.

In oceans, sediments are one of the most-studied marine sources for actinobacterial isolation [8,9]. Previous work in the Cantabrian Sea (Biscay Bay, Northeast Atlantic), have revealed that bioactive actinobacteria—mainly *Streptomyces* species—are associated to corals and other invertebrates living up to 4700 m depth in the submarine Avilés Canyon [10,11,12]. Actinobacteria displaying a wide repertoire of chemically diverse secondary metabolites with different antibiotic or antitumor activities have been isolated from coral reefs ecosystems from the Avilés Canyon [13]. Recently, new natural products with antibiotic and cytotoxic activities have been reported in this Canyon [14,15]. Paulomycins A and B have been reported to be produced by a ubiquitous *Streptomyces albidoflavus* strain widely distributed among terrestrial, marine, and atmospheric environments in the Cantabrian Cornice [12].

Herein, we report the discovery of a novel natural product, paulomycin G (**1**), obtained from *Micromonospora matsumotoense* M-412, isolated from deep sea sediments collected at 2000 m depth during an oceanographic expedition to the submarine Avilés Canyon. The presence of a new paulomycin not previously reported was identified in the extract by LC-UV-MS and LC-HRMS chemical dereplication [16], and further efforts were focused on the isolation, structural elucidation, and biological properties of this new molecule. Paulomycin G is also the first member of the family displaying strong cytotoxic activity against different human tumour cell lines, such as pancreatic adenocarcinoma (MiaPaca_2), breast adenocarcinoma (MCF-7), and hepatocellular carcinoma (HepG2).

## 2. Results and Discussion

### 2.1. Taxonomy and Phylogenetic Analysis of the Strain

**Figure 1:**
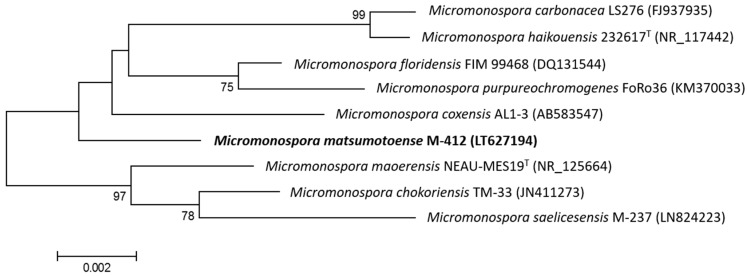
Neighbour-joining phylogenetic tree obtained by distance matrix analysis of 16S rDNA sequences, showing *Micromonospora matsumotoense* M-412 position and most closely related phylogenetic neighbours. Numbers on branch nodes are bootstrap values (1000 resamplings; only values >70% are given). Bar indicates 0.2% sequence divergence.

The 16S rDNA of producing strain M-412 was amplified by polymerase chain reaction (PCR) and sequenced. After Basic Logic Alignment Search Tool (BLAST) sequence comparison, strain M-412 showed 100% identity to *Micromonospora matsumotoense* (Accession number NR_025015); thus, this strain was designated as *Micromonospora matsumotoense* M-412 (EMBL Sequence number LT627194). The phylogenetic tree generated by a neighbour-joining method based on 16S rDNA sequence clearly revealed the evolutionary relationship of strain M-412 with a group of known *Micromonospora* species (Figure 1). To our knowledge, all known paulomycin compounds have only been produced by *Streptomyces*; thus, paulomycin G is the first member of the family produced by a *Micromonospora* species.

### 2.2. Structure Determination

**Figure 2:**
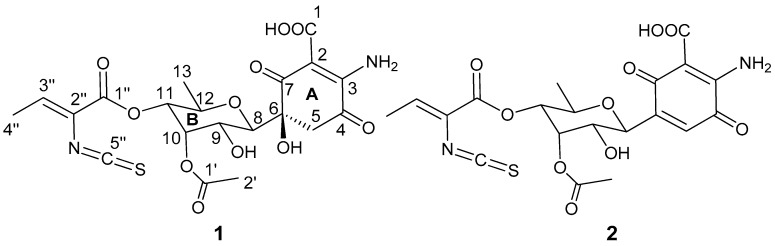
Chemical structures of paulomycin G (**1**) and compound **2**.

Compound **1** was isolated as a pale yellow solid. LC-UV-HRMS analysis of the isolated sample revealed a purity of 83.5% (UV at 210 nm), and indicated the presence of a major impurity in the sample (16.5%), which was identified as the dehydrated paulomycinone **2** based on its UV and HRMS spectra (see Figures S1, S4 and S5). Dehydration of paulomycins has been described to occur easily, even when leaving the compounds in solution in aqueous media at neutral pH, and is therefore difficult to avoid [17]. The major compound of the mixture, paulomycin G, had a molecular formula of C_20_H_22_N_2_O_11_S according to ESI-TOF MS measurements (*m*/*z* 499.1015 [M + H]^+^, calcd. for C_20_H_23_N_2_O_11_S^+^, 499.1017) and the presence of 20 signals in its ^13^C NMR spectrum. Its UV spectrum displayed maxima at 238, 276, and 320 nm, in agreement with a paulomycin-like structure [2,3]. An intense absorption band at 2041 cm^−1^ in its IR spectrum confirmed the presence of an isothiocyanate group in the molecule, present in the pauloate moiety of all paulomycins. NMR spectra (Table 1) confirmed the presence of this pauloate moiety, with signals for a methyl group (δ_H_ 1.89, δ_C_ 14.6 ppm) coupled to an sp^2^ proton (δ_H_ 6.71, δ_C_ 136.9 ppm) in the COSY spectrum. HMBC correlations from the latter proton to carbons at δ_C_ 159.8 (α,β-unsaturated carbonyl group C1′′), 122.3 ppm (sp^2^ quaternary carbon C2′′), 141.6 ppm (isothiocyanate carbon C5′′, four-bond distance correlation), and 14.6 ppm (methyl C4′′) completed the structural assignment of the pauloate moiety. Additionally, signals for an aliphatic methylene at δ_H_ 3.22 and 3.17 that correlated in the HMBC spectrum with carbon signals at δ_C_ 159.4 (C3), 188.8 (C4), 77.5 (C6 and C8), and 197.5 (C7), and the presence of two additional signals at δ_C_ 169.0 and 99.1 ppm accounted for the presence in the molecule of the 6-substituted 2-amino-5-hydroxy-3,6-dioxocyclohex-1-enecarboxylic acid substructure present in the paulomycin family of compounds. On the other hand, signals for five oxygenated methines at δ_H_ 3.69, 3.61, 5.29, 4.51, and 3.79 ppm, and one aliphatic methyl group at 0.88 ppm conformed a spin system according to COSY correlations that accounted for ring B in the structure of the molecule. Connection between carbon C6 of ring A and carbon C8 in ring B was additionally confirmed via HMBC correlations observed between H8 and C5, C6, and C7. Finally, signals for an acetyl functionality were observed (δ_H_ 2.10 ppm, δ_C_ 170.1, and 20.8 ppm). This acetyl group was placed at C10 based on HMBC correlations of H2′ and H10 to the carbonyl carbon C1′ and the low field chemical shift of H10. The pauloate moiety was similarly placed at C11 based on an HMBC correlation observed between H11 and C1′′. The relative configuration proposed around the chiral centres in ring B was based on the large coupling constants observed between H8 and H9 (9.8 Hz) and between H11 and H12 (9.9 Hz), indicating the axial orientation of all these protons. The two small coupling constants measured for H10 strongly suggested an equatorial orientation for this proton, and finally, correlations observed in the ROESY spectrum between H10, H9, and H11, and between H8 and H12, proved a relative configuration for ring B as depicted in Figure 2. Based on biogenetic considerations, the absolute configuration was assumed to be the same as in other compounds of the paulomycin series.

Paulomycin G is a novel natural product, structurally unique since it constitutes the first member of the paulomycin family of antibiotics lacking the paulomycose moiety and having a methyl group at position C-13. It is also the smallest bioactive paulomycin reported to-date. 

### 2.3. Cytotoxic Activity of Paulomycin G

**Figure 3:**
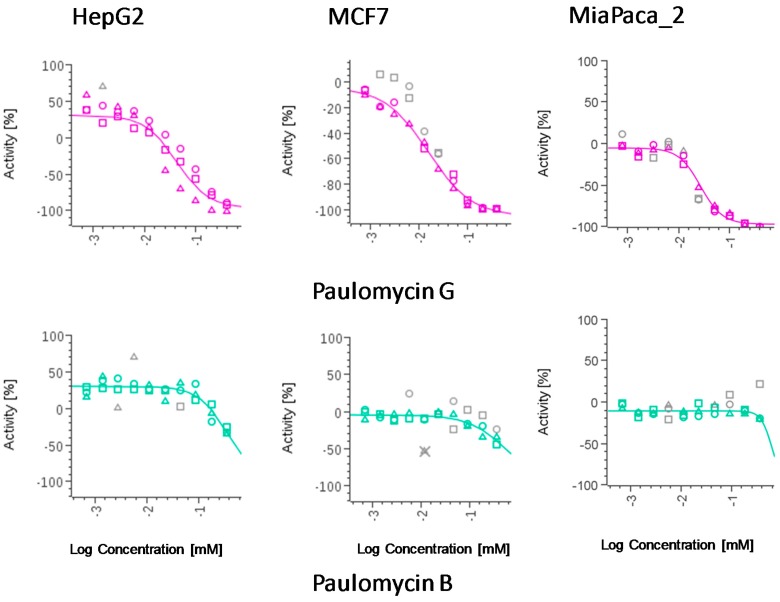
Dose-response curves of compounds against human breast adenocarcinoma (MCF-7), pancreatic adenocarcinoma (MiaPaca_2), and hepatocellular carcinoma (HepG2) cell lines. Compounds were tested per triplicate and the obtained results are indicated with triangles, rounds, and squares in every picture.

Cytotoxic activity was observed for the compound against human breast adenocarcinoma (MCF-7), pancreatic adenocarcinoma (MiaPaca_2), and hepatocellular carcinoma (HepG2) cell lines (Table 2). Considering the lack of biological activity reported for compounds having the quinone moiety present in compound **2** [18], it is reasonable to assume that the biological activity reported herein for the isolated sample is mostly due to paulomycin G. Paulomycin B did not display any cytotoxic activity against the three cell lines when tested in parallel. Figure 3 represents the dose-response curves of paulomycins B and G against the different tumour cell lines.

### 2.4. Antimicrobial Activity of Paulomycin G

The antimicrobial activity of Paulomycin G was tested against a panel of pathogenic bacteria and fungi, including Gram-negative (*Pseudomonas aeruginosa*, *Acinetobacter baumannii*, *Escherichia coli*, and *Klebsiella pneumoniae*) and Gram-positive bacteria (methicillin-resistant *Staphylococcus aureus*, MRSA) and fungi (*Aspergillus fumigatus* and *Candida albicans*). Paulomycin B was tested in parallel against the same panel of pathogens. One of the *E. coli* strains tested (MB5746) was the only pathogen whose growth was inhibited by the action of paulomycin G, with an MIC_90_ of 38 μg/mL. Paulomycin B displayed activity against *E. coli* MB5746 and MRSA, with MIC_90_ values of 4.5 and 50 μg/mL, respectively.

## 3. Materials and Methods 

### 3.1. General Experimental Procedures

Semipreparative HPLC was performed with an Alliance chromatographic system (Waters Corporation, Mildford, MA, USA) and an Atlantis C18 column (10 μm, 10 × 150 mm, Waters). For UPLC analysis an Acquity UPLC equipment (Waters) with a BEH C18 column (1.7 μm, 2.1 × 100 mm, Waters) was used. Optical rotations were determined with a JASCO P-2000 polarimeter (JASCO Corporation, Tokyo, Japan). IR spectrum was measured with a JASCO Fourier transform infrared (FT/IR)-4100 spectrometer (JASCO Corporation) equipped with a PIKE MIRacle^TM^ single reflection ATR accessory. NMR spectra were recorded on a Bruker Avance III spectrometer (500 and 125 MHz for ^1^H and ^13^C NMR, respectively) equipped with a 1.7 mm TCI MicroCryoProbe^TM^ (Bruker Biospin, Fällanden, Switzerland), using the signal of the residual solvent as internal reference (δ_H_ 2.50 and δ_C_ 39.5 ppm for DMSO-*d*_6_). ESI-TOF MS spectra were acquired with a Bruker maXis QTOF spectrometer (Bruker Daltonik GmbH, Bremen, Germany).

### 3.2. Microorganism and Fermentation Conditions

Strain M-412 was isolated from a deep-sea sediment sample collected from the Cantabrian Sea at a depth of 2000 m, as previously described [13]. GHSA medium (1% glucose, 1% soy bean flour, 0.05% yeast extract, 2.1% MOPS, 0.06% MgSO_4_·7H_2_O, 0.2% of a trace elements solution from R5A medium [19], pH 6.8) was selected for paulomycin G production. After autoclaving, the medium was supplemented with 0.4% of a 5 M solution CaCl_2_·2H_2_O and 3% DMSO. 20 Erlenmeyer flasks (250 mL), each containing 50 mL of GHSA medium, were inoculated with spores and incubated in an orbital shaker at 28 °C and 250 rpm during 7 days. 

### 3.3. Isolation and Purification of Paulomycin G

The culture broths were centrifuged, and the pellets were extracted with ethyl acetate acidified with 1% formic acid. The supernatants were filtered and applied to a solid-phase extraction cartridge (Sep-Pak Vac C18, 10 g, Waters) that was eluted using a gradient of methanol and 0.05% TFA in water from 0 to 100% methanol in 60 min, at a flow rate of 5 mL/min. Fractions were collected every 5 min and analysed by UPLC using chromatographic conditions previously described [5]. A peak corresponding to an unknown paulomycin was detected in fractions eluting between 40 and 50 min. These fractions were pooled, partially dried in vacuo, and applied to a solid-phase extraction cartridge (Sep-Pak Vac C18, 2 g, Waters). The cartridge was washed with water and the retained compounds were eluted with methanol and dried in vacuo. The residue was subsequently redissolved in a small volume of acetonitrile and DMSO (2:1). The same peak of unknown paulomycin was also found in the organic extract of the culture pellets, which was dried and redissolved as above. Paulomycin G was eventually purified by semipreparative HPLC using an Atlantis C18 column (10 μm, 10 × 150 mm, Waters) in two isocratic elution steps, employing a mixture of 50% acetonitrile and water in the first step and 55% methanol and water in the second step, with a flow of 5 mL/min. In both cases, the solution containing the collected peak was evaporated and finally lyophilized, resulting in 2.7 mg of compound **1** (83.5% purity according to LC-UV analysis at 210 nm).

Paulomycin G (**1**). pale yellow solid; ${\left[\alpha \right]}_{D}^{20}$ +10.6° (*c* 0.18, MeOH); UV (DAD) *λ*_max_ 238, 276 y 320 nm; IR (ATR) *ν*_max_ 3359, 3229, 2979, 2933, 2041, 1736, 1695, 1626, 1571, 1442, 1381, 1260, 1227, 1129, 1025, 909, 751 cm^−1^; for ^1^H and ^13^C NMR data see Table 1; ESI-TOF MS *m*/*z* 521.0828 [M + Na]^+^ (calcd. for C_20_H_22_N_2_O_11_SNa^+^, 521.0837) 516.1278 [M + NH_4_]^+^ (calcd. for C_20_H_26_N_3_O_11_S^+^, 516.1283), 499.1015 [M + H]^+^ (calcd. C_20_H_23_N_2_O_11_S^+^, 499.1017).

### 3.4. Phylogenetic Analysis (Taxonomy) of the Producer Microorganism

Phylogenetic analysis based on 16S rRNA sequences was performed with strain *Micromonospora matsumotoense* M-412 using MEGA version 6.0 [20] after multiple alignment of data by Clustal Omega [21]. Distances (distance options according to the Kimura two-parameter model [22]) and clustering with the neighbour-joining method [23] were evaluated using bootstrap values based on 1000 replications [24].

### 3.5. Cytotoxic Activity of Compound ***1***

The MTT (3-(4,5-dimethylthiazol-2-yl)-2,5-diphenyltetrazolium bromide) colorimetric assay—which measures mitochondrial metabolic activity—was performed using three tumour cell lines obtained from the ATCC, namely human breast adenocarcinoma (MCF-7), pancreatic adenocarcinoma (MiaPaca_2), and hepatocellular carcinoma (HepG2), using previously-described methodology [15]. Methyl methanesulphonate at a concentration of 8 mM and 0.5% DMSO in water were used as positive and negative controls, respectively.

### 3.6. Antimicrobial Activity of Compound ***1***

Antibacterial and antifungal activity tests of paulomycins G and B were performed against the pathogenic strains *P. aeruginosa* PAO1, *Acinetobacter baumannii* MB5973, *Escherichia coli* MB5746 and MB2884, *K. pneumoniae* ATCC700603, methicillin-resistant *Staphylococcus aureus* MB5393, *Aspergillus fumigatus* ATCC46645, and *Candida albicans* ATCC64124, as previously described [25].

## 4. Conclusions

In summary, a new member of the paulomycin family that we have designated as paulomycin G has been obtained from cultures of *Micromonospora matsumotoense* M-412, isolated from deep-sea sediments collected at 2000 m depth in the submarine Avilés Canyon. Paulomycin G is a novel natural product, structurally unique since—to our knowledge—it is the first member of the paulomycin family of antibiotics lacking the paulomycose moiety, being the smallest bioactive paulomycin reported. This new natural product displayed strong cytotoxic activity against human cancer cell lines such as pancreatic adenocarcinoma (MiaPaca_2), breast adenocarcinoma (MCF-7), and hepatocellular carcinoma (HepG2). Based on its cytotoxic activities, paulomycin G deserves to be considered as a candidate to perform further studies assessing its anticancer potential. Besides its unique structural features, paulomycin G might also be of interest in biosynthetic studies and useful for future paulomycin biosynthesis research or as a core structure in the generation of novel derivatives through combinatorial biosynthesis to generate structural diversity in the paulomycin family. Additionally, the isolation of a compound of the paulomycin family from a non-*Streptomyces* actinomycete is particularly noteworthy and suggests horizontal gene transfer between *Micromonospora* and *Streptomyces* species.

## Figures and Tables

**Table 1:** ^1^H and ^13^C NMR (500 and 125 MHz in DMSO-*d*_6_) data for compound **1**.

Position	δ ^13^C	δ (^1^H), (Mult, *J* in Hz)
1	169.0	-
2	99.1	-
3	159.4	-
4	188.8	-
5	47.7	3.22 (d, 16.0), 3.17 (d, 16.0)
6	77.5	-
7	197.5	-
8	77.5	3.69 (d, 9.9)
9	67.0	3.61 (br dt, 9.7, 3.3)
10	69.8	5.29 (dd, 2.6, 2.6)
11	73.4	4.51 (dd, 9.9, 2.6)
12	69.9	3.79 (dq, 9.9, 6.2)
13	16.3	0.88 (d, 6.2)
1′	170.1	-
2′	20.8	2.10 (s)
1′	159.8	-
2′′	122.3	-
3′′	136.9	6.71 (quart., 7.1)
4′′	14.6	1.89 (d, 7.1)
5′′	141.6	-
NH_2_ (3)	-	9.71 (br s), 9.35 (br s)
OH (1)	-	14.21 (br s)
OH (6)	-	5.43 (s)
OH (9)	-	5.78 (d, 4.4)

**Table 2:** Cytotoxic activity of paulomycins B and G against different tumour cell lines.

Cell line	Paulomycin G (IC_50_ μM)	Paulomycin B (IC_50_ μM)
HepG2	4.30 ± 0.42	>36
MCF-7	1.58 ± 0.12	>36
MiaPaca_2	2.70 ± 0.25	>36

## Supplementary Materials

The following are available online at www.mdpi.com/1660-3397/15/9/271/s1. Figure S1: HPLC trace of the sample isolated; Figure S2: UV spectrum of compound 1; Figure S3: ESI-TOF spectra of compound **1**; Figure S4: UV spectrum of compound **2**; Figure S5: ESI-TOF spectra of compound **2**; Figure S6: ^1^H NMR spectrum (DMSO-*d*6, 500 MHz) of compound **1**; Figure S7: ^13^C NMR spectrum (DMSO-*d*6, 125 MHz) of compound **1**; Figure S8: COSY spectrum (DMSO-*d*6) of compound **1**; Figure S9: HSQC spectrum (DMSO-*d*6) of compound **1**; Figure S10: HMBC spectrum (DMSO-*d*6) of compound **1**; Figure S11: ROESY spectrum (DMSO-*d*6) of compound **1**; Figure S12: Picture of Micromonospora matsumotoense M-412.
