# Supplementary material for: Paulomycin G, a New Natural Product with Cytotoxic Activity against Tumor Cell Lines Produced by Deep-Sea Sediment Derived Micromonospora matsumotoense M-412 from the Avilés Canyon in the Cantabrian Sea
Source: Mar Drugs. 2017 Aug 28;15(9):271. doi: 10.3390/md15090271 (PMC5618410; doi:10.3390/md15090271)
Supplement: Supplementary file 1 [file marinedrugs-15-00271-s001.pdf]

Paulomycin G, a new natural product with cytotoxic activity against tumor cell lines produced by a deep-sea sediment derived *Micromonospora matsumotoense* M-412 from the Avilés Canyon in the Cantabrian Sea

Aida Sarmiento-Vizcaíno[1], Alfredo F. Braña[1], Ignacio Pérez-Victoria[2], Jesús Martín[2], Nuria de Pedro[2], Mercedes de la Cruz[2], Caridad Díaz[2], Francisca Vicente[2], José L. Acuña[3], Fernando Reyes[2]*, Luis A. García[4] and Gloria Blanco[1]*

List of supplementary materials

Figure S1. HPLC trace of the sample isolated.

Figure S2. UV spectrum of compound **1**.

Figure S3. ESI-TOF spectra of compound **1**.

Figure S4. UV spectrum of compound **2**.

Figure S5. ESI-TOF spectra of compound **2**.

Figure S6. ^{1}H NMR spectrum (DMSO-d_6, 500 MHz) of compound **1**.

Figure S7. ^{13}C NMR spectrum (DMSO-d_6, 125 MHz) of compound **1**.

Figure S8. COSY spectrum (DMSO-d_6) of compound **1**.

Figure S9. HSQC spectrum (DMSO-d_6) of compound **1**.

Figure S10. HMBC spectrum (DMSO-d_6) of compound **1**.

Figure S11. ROESY spectrum (DMSO-d_6) of compound **1**.

Figure S12. Picture of *Micromonospora matsumotoense* M-412.

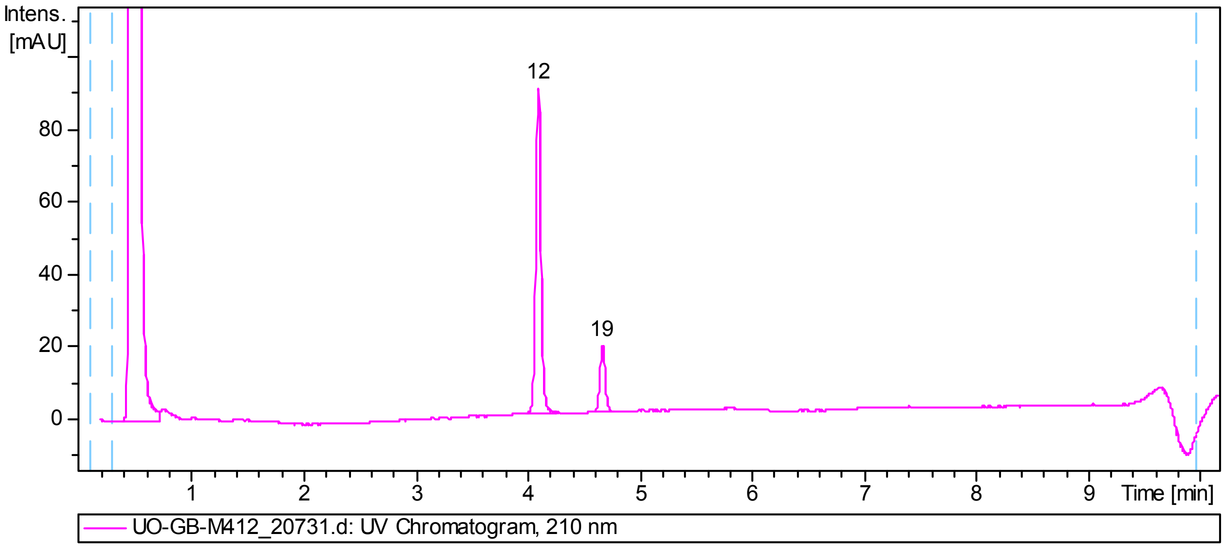

Compound	Peak	RT [min]	Area	% Area
**1**	12	4.10	293	83.5
**2**	19	4.66	58	16.5

Figure S1. HPLC trace of the sample isolated.

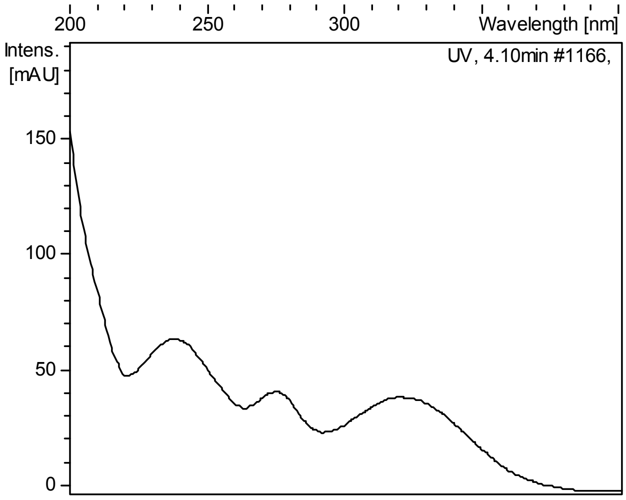

Maxima: 238, 276 and 320nm

Figure S2. UV spectrum of compound **1**.

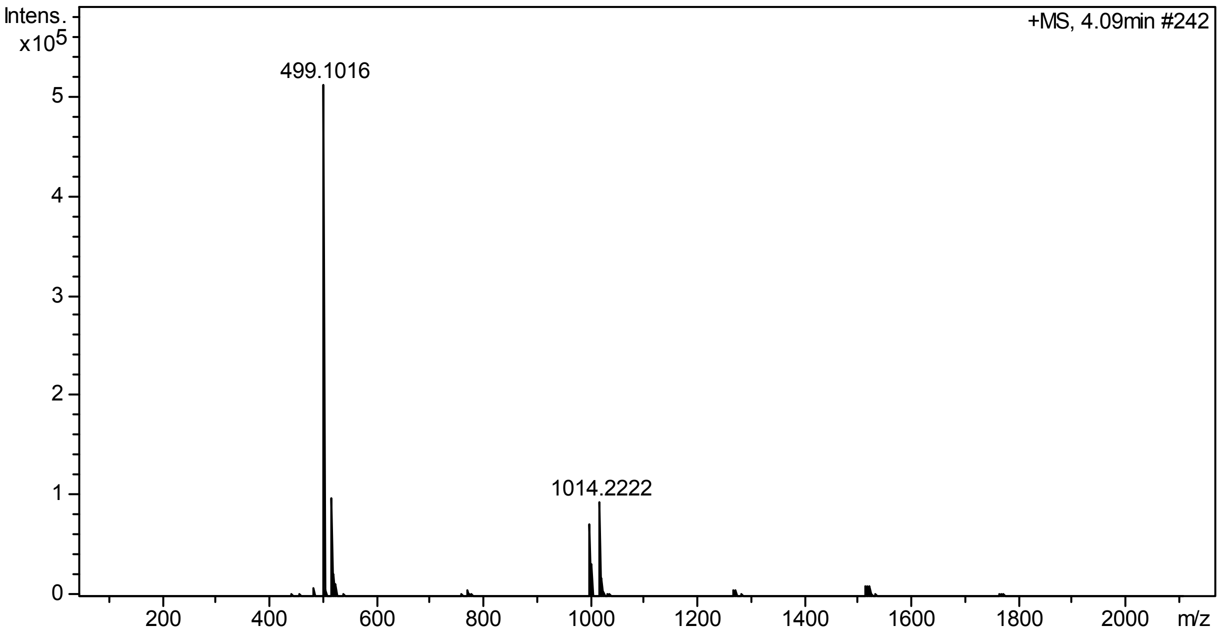

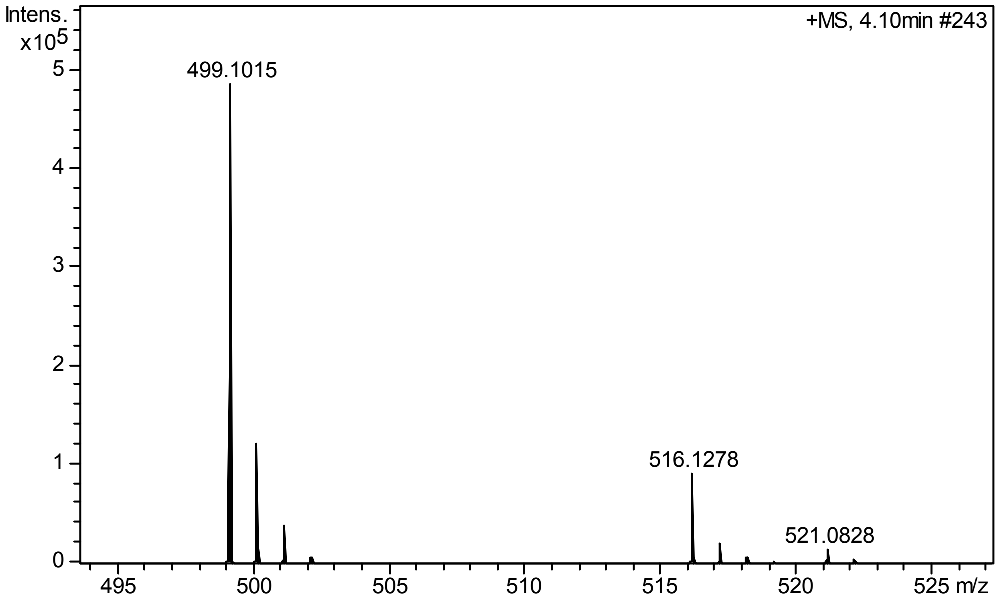

$C_{20}H_{23}N_2O_{11}S^+$: calc= 499.101707; exp= 499.1015; err=-0.4 ppm
$C_{20}H_{26}N_3O_{11}S^+$: calc= 516.128256; exp=516.1278; err=-0.9 ppm
$C_{20}H_{22}N_2O_{11}SNa^+$: calc= 521.083651; exp=521.0828; err=-1.6 ppm

Figure S3. ESI TOF spectra of compound **1**.

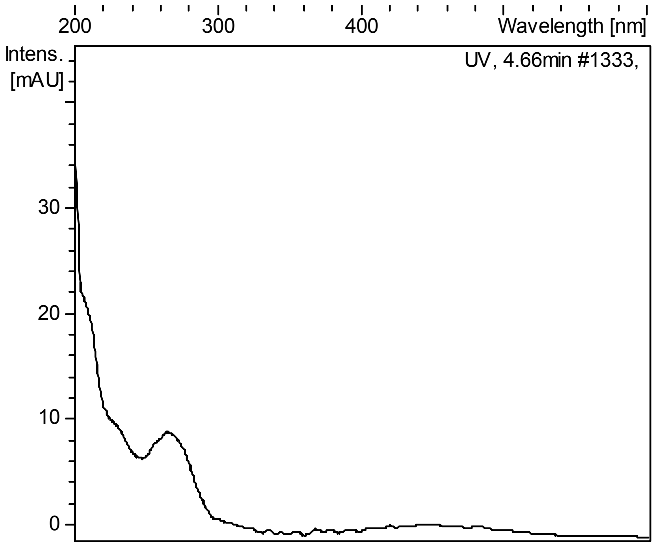

Figure S4. UV spectrum of compound **2**.

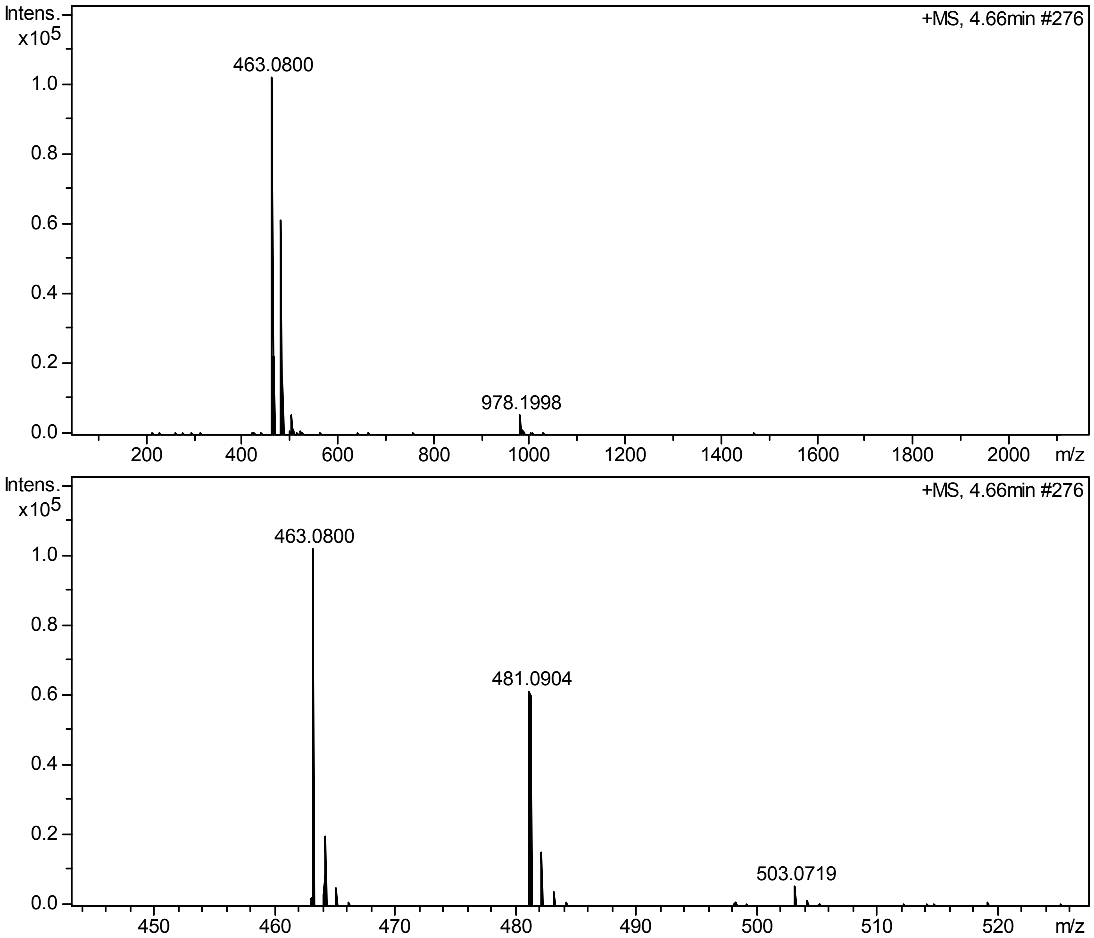

$C_{20}H_{21}N_2O_{10}S^+$: calc= 481.091142; exp= 481.0904; err=-1.5 ppm
$C_{20}H_{19}N_2O_9S^+$: calc= 463.080577; exp=463.0800; err=-1.2 ppm
$C_{20}H_{22}N_2O_{10}SNa^+$: calc= 503.073087; exp=503.0719; err=-2.4 ppm

Figure S5. ESI TOF spectra of compound **2**.

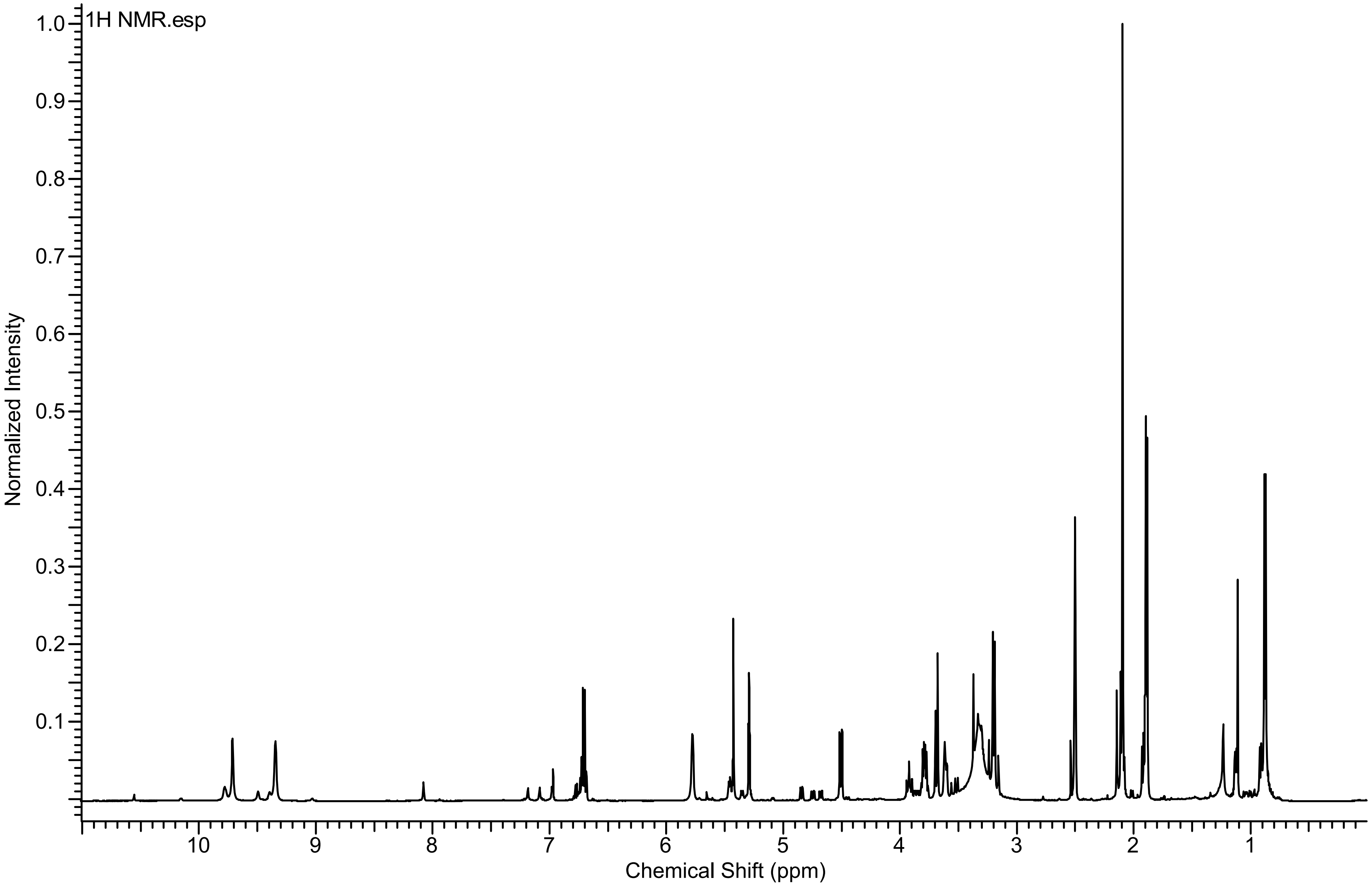

Figure S6. ^{1}H NMR (DMSO-d_6, 500 MHz) of compound **1**.

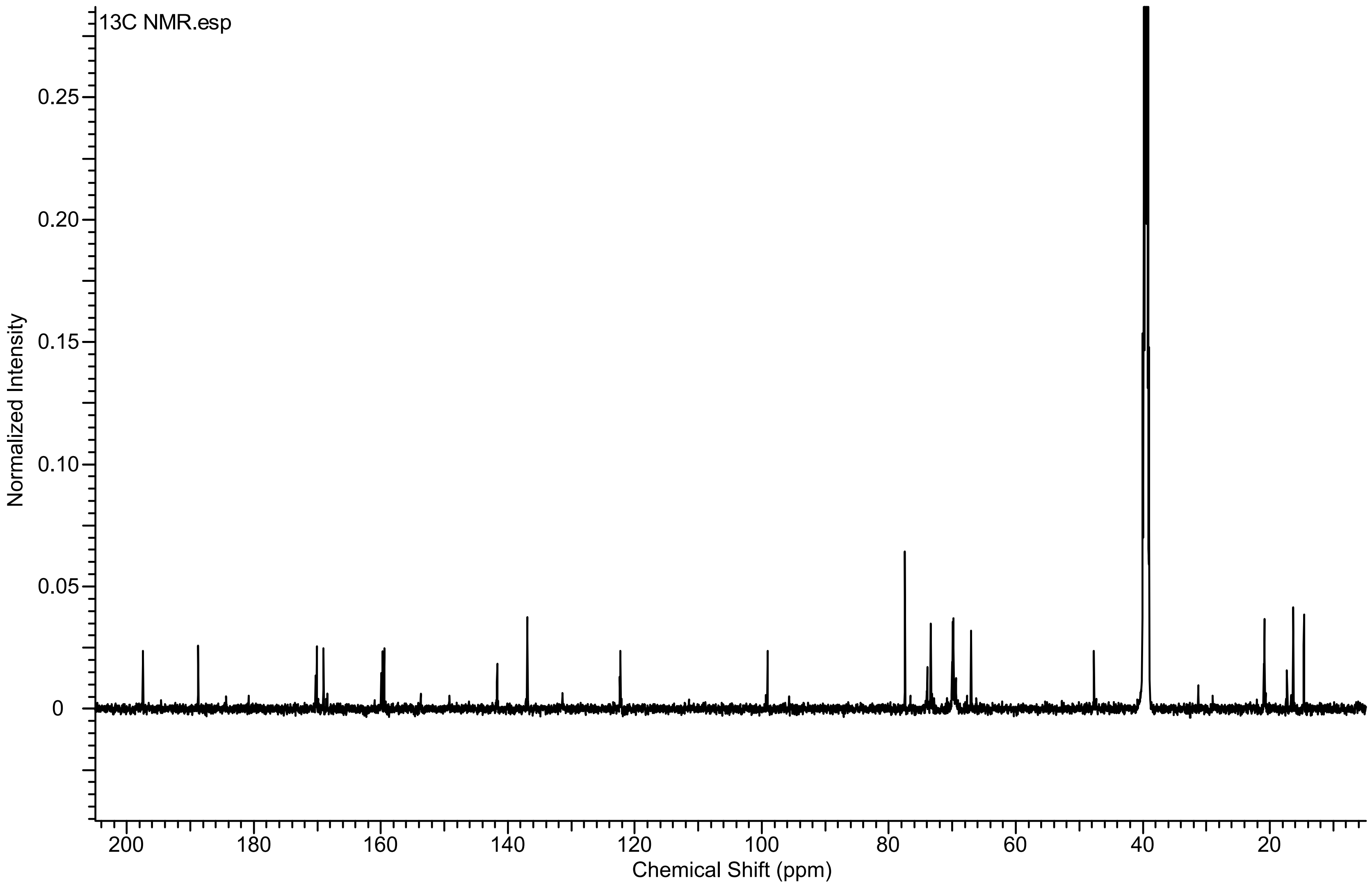

Figure S7. ^{13}C NMR (DMSO-d_6, 125 MHz) of compound **1**.

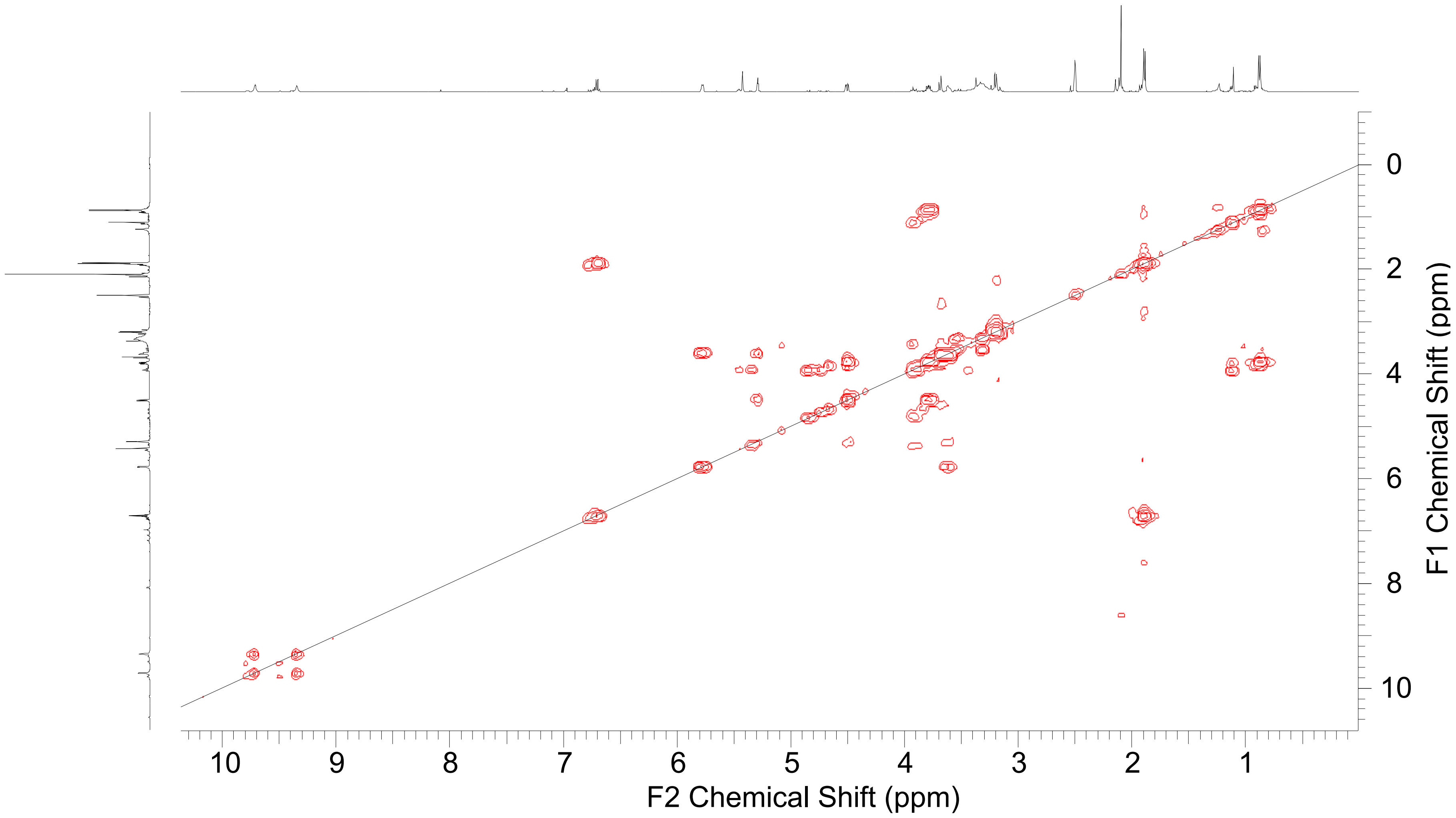

Figure S8. COSY spectrum (DMSO-d_6) of compound **1**.

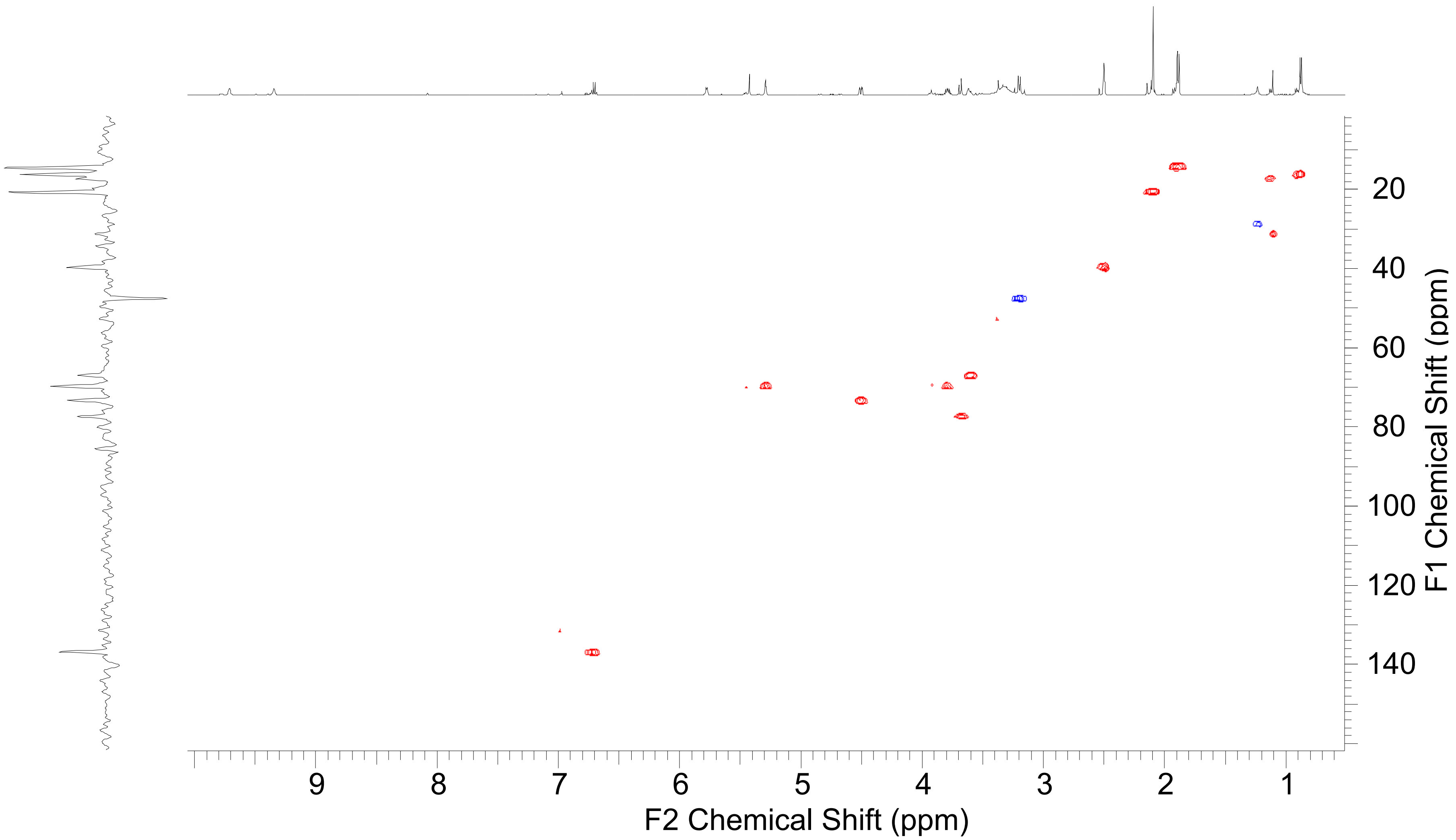

Figure S9. HSQC spectrum (DMSO-d_6) of compound **1**.

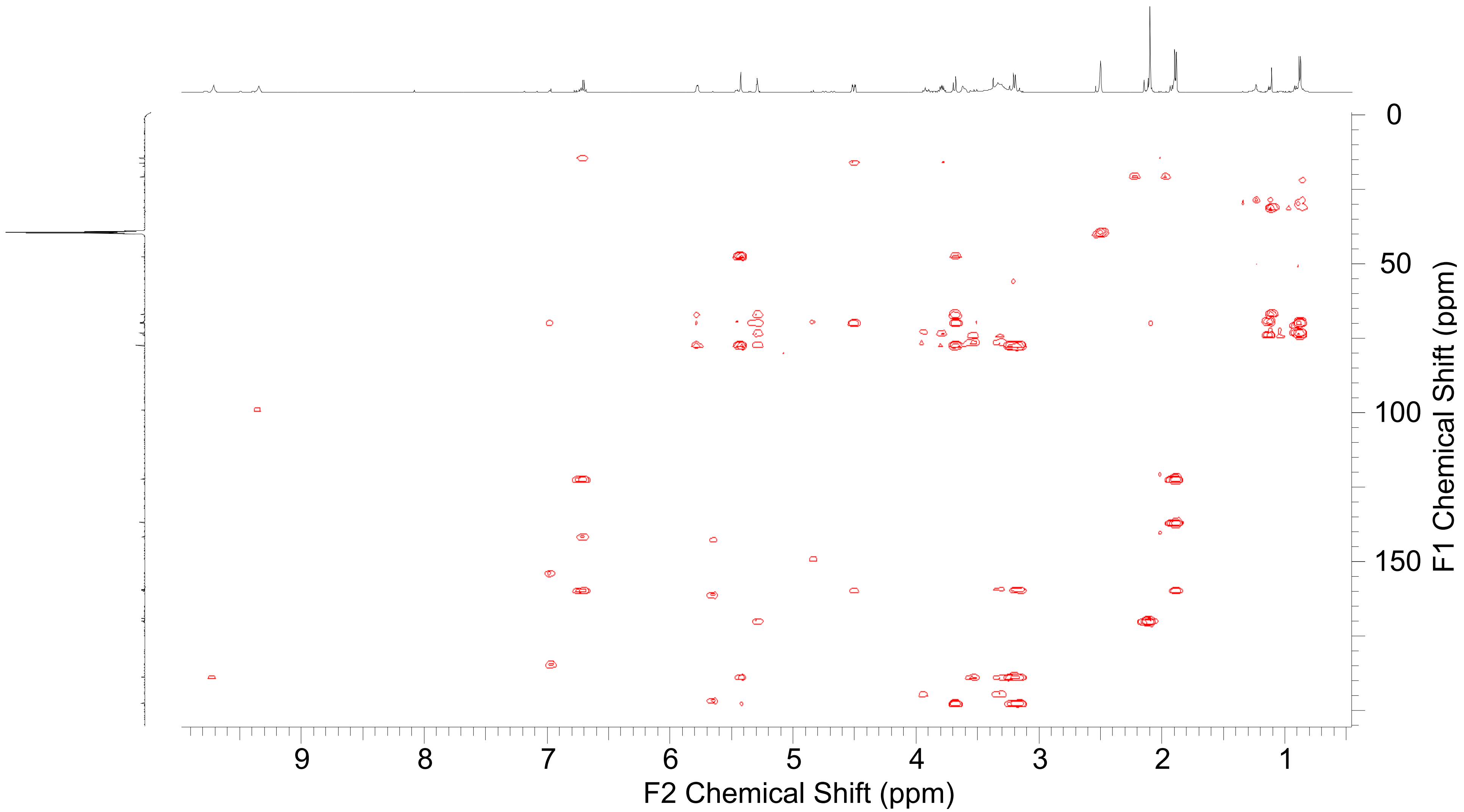

Figure S10. HMBC spectrum (DMSO-d_6) of compound **1**.

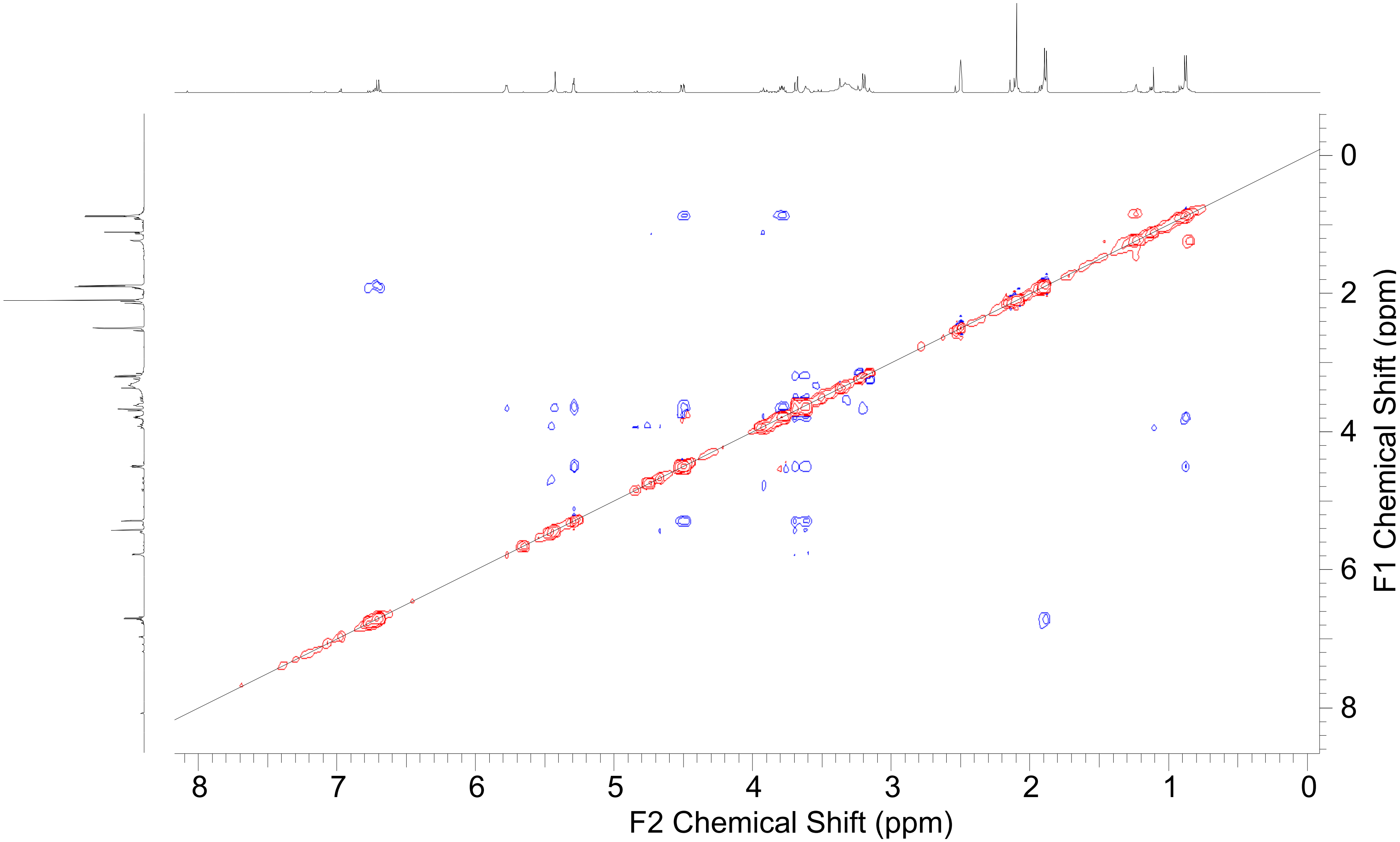

Figure S11. ROESY spectrum (DMSO-d_6) of compound **1**

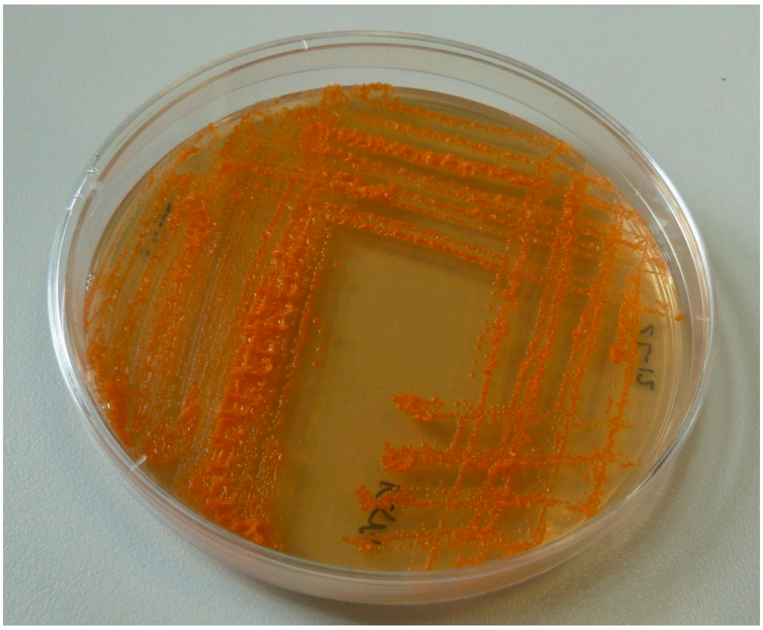

Figure S12. Picture of *Micromonospora matsumotoense* M-412.
